# The Management of Combined Antithrombotic Therapy in Patients With Atrial Fibrillation Undergoing Percutaneous Coronary Intervention: A Particularly Complex Challenge, Especially in the Elderly

**DOI:** 10.3389/fphys.2018.00876

**Published:** 2018-07-09

**Authors:** Leonardo Bencivenga, Klara Komici, Graziamaria Corbi, Antonio Cittadini, Nicola Ferrara, Giuseppe Rengo

**Affiliations:** ^1^Department of Translational Medical Sciences, University of Naples Federico II, Naples, Italy; ^2^Department of Medicine and Health Sciences, University of Molise, Campobasso, Italy; ^3^Istituti Clinici Scientifici Maugeri SpA Società Benefit (ICS Maugeri SpA SB), Telese Terme, Italy

**Keywords:** antithrombotic therapy, anticoagulation, antiplatelet, atrial fibrillation, percutaneous coronary intervention, elderly, frailty

## Abstract

Anticoagulation is superior to dual antiplatelet therapy (DAPT) in the prevention of thromboembolic events in patients with atrial fibrillation (AF), otherwise the prevention of ischemic risk and stent thrombosis after percutaneous coronary intervention (PCI) is warranted by DAPT. The coexistence of conditions requiring combined antithrombotic therapies is becoming an increasing relevant clinical problem, whose therapeutic management has long been found in the medical experience rather than in guidelines and consensus. Recently, updates in guidelines and relevant studies have been published in order to better clarify the best management of combined antithrombotic therapy. In the present review, we have analyzed the recent literature, underlining the progresses and the residual limits of the most up-to-date evidence on the management of patients requiring dual or triple antithrombotic therapy, due to coexisting AF and coronary artery disease. An in-depth overview is also focused on the management of antithrombotic therapy in the elderly patient, which represents an even more complex challenge for the clinician. This is due to the high prevalence, among over 65 years aged people, of conditions requiring association of antiplatelet and anticoagulant drugs, the numerous comorbidities, the higher risk of complications, such as bleedings, and the lack of specific evidence, especially for the frail elderly. Nowadays, triple therapy [oral anticoagulation (OAC) plus dual antiplatelet agents] for the shortest possible time should be the treatment for AF patients undergoing PCI, whereas dual therapy (single antiplatelet plus OAC) may be preferred for patients at high bleeding risk.

## Introduction

The combination of antiplatelet and anticoagulant therapies is required in many conditions of great clinical impact, especially among the over 65 years aged people. AF, the most frequent arrhythmia, whose prevalence is age-related (Chugh et al., 2014), coexists in a large proportion of patients with CAD. The therapeutic management of concomitant cardiovascular diseases is even more insidious in a population susceptible to falls and related complications, such as femoral fractures, and high risk of hemorrhage. Moreover, treatment of cardiovascular diseases in pre-frail and frail elderly patients is extremely complex because recommended standard therapies cannot often be applied in these people (Bencivenga et al., 2017). In this context, especially in the frail elderly patients, there are more and more frequent evidence of under prescription of antithrombotic therapies due to the mentioned difficulties, which lead the physician to be more restrained by fear of complications of these treatments, rather than reassured by evidence to their actual necessity, though stated in the current guidelines (Hess et al., 2012). However, despite advanced age represents a non-modifiable bleeding risk factor, geriatric population presents increased thromboembolic risk and antithrombotic therapies are even more effective, on mortality and ischemic stroke rates, in patients aged 65 years and over (Mant et al., 2007). Besides, regarding the most recommended bleeding risk predictive models developed for patients on OAC therapy, such as HAS-BLED, advanced age represents *per se* a risk factor, frequently complicated by additional ones. Indeed, isolated systolic hypertension, anemia, pervious stroke or hemorrhage, impaired renal and liver function are very common in people aged >65 years, which are also the main users of antiplatelet drugs and non-steroidal anti-inflammatory drug (Kirchner, 1994), that contribute to increase bleeding risk. Furthermore, none of the scores for anticoagulated patients has been tested in prospective randomized controlled trials (Kirchhof et al., 2016). Anyhow, among the main influencing factors, “age” is the only one included in all the predictive scores of thrombotic or hemorrhagic risk (Kirchhof et al., 2016).

The problem of how to handle double (single antiplatelet plus OAC) or triple (OAC plus dual antiplatelet agents) antithrombotic therapy is raising a great interest in the scientific community. Indeed, the most recent European guidelines on the therapeutic management of thromboembolic risk in patients with AF dedicate an entire section to the management of patients with associated ACS, under medical therapy or undergoing PCI (Kirchhof et al., 2016); although not directly designed on geriatric populations, the evidence that allowed to define a therapeutic flow-chart derives from studies on populations with an average age of over 65 years (Sarafoff et al., 2013; Braun et al., 2015). Finally, in a recent focused update from the ESC on DAPT in patients with CAD, a specific paragraph has been dedicated to subpopulation requiring concomitant anticoagulant therapy, argued on the basis of trials on elderly populations (Valgimigli et al., 2018).

## Therapeutic Recommendations in Patients With Atrial Fibrillation and Coronary Artery Disease

Atrial fibrillation represents the most common arrhythmia, whose prevalence significantly increases with age (Wilke et al., 2013); its incidence is also rapidly growing outlining a global epidemic with tremendous burden of disability and mortality worldwide (Chugh et al., 2014). The incidence of CAD in patients with AF is very high (Kralev et al., 2011) and it is estimated that up to 7% of patients undergoing PCI for CAD suffer of AF or have another indication for OAC (Alexopoulos et al., 2017). Furthermore, AF represents a frequent complication in patients with acute myocardial infarction (AMI) (Ibanez et al., 2017), contributing to worsening prognosis, whereas advanced age and heart failure constitute the main predictors for the onset of this arrhythmia in AMI patients (Schmitt et al., 2009).

According to the most recent ESC guidelines for AF, an indication for OAC therapy subsists in all patients with paroxysmal, persistent or permanent AF presenting a thromboembolic risk assessed by CHA_2_DS_2_-VASc score (≥2 in men and ≥3 in women). The CHA_2_DS_2_-VASc is a score widely validated for the prediction of the thromboembolic risk in AF patients; it ranges from 0 to 9 and attributes 1 point for age between 65 and 74 years, female gender, presence of congestive heart failure/left ventricular dysfunction, hypertension, diabetes, vascular disease, and 2 points for age ≥75 years and history of previous stroke or thromboembolism (Kirchhof et al., 2016). OAC therapy has demonstrated a significant positive effect on ischemic stroke prevention and mortality rates, in particular among the elderly population, as mentioned above (Mant et al., 2007). Warfarin, a VKA, represents the most used OAC, but its management is complicated by several drug and food interactions, narrow therapeutic range (INR 2–3 in AF) for thromboembolic prevention, which requires frequent laboratory monitoring and dose adjustments (Sharma et al., 2015). Non-VKA anticoagulants, known as new oral anticoagulants (NOACs), include factor Xa inhibitors (rivaroxaban, apixaban, and edoxaban) and direct thrombin inhibitor (dabigatran). In the last decade, several clinical trials have demonstrated that NOACs are non-inferior [RE-LY: dabigatran vs. warfarin (Connolly et al., 2009), ROCKET-AF: rivaroxaban vs. warfarin (Patel et al., 2011), ENGAGE-TIMI: edoxaban vs. warfarin (Giugliano et al., 2013)] or even superior [ARISTOTLE: apixaban vs. warfarin (Granger et al., 2011)] to VKA in preventing stroke or thromboembolism, with a similar or inferior risk of major bleeding. Furthermore, they overcome many of the mentioned disadvantages of VKA. According to the ESC guidelines for AF, a NOAC should be preferred to VKA when starting OAC therapy in non-valvular AF (NVAF) patients (Kirchhof et al., 2016).

Dual antiplatelet therapy represents the essential treatment in patient with stable CAD undergoing stenting after PCI: clopidogrel (P2Y_12_ receptor antagonist) in addition to aspirin lifelong is recommended for 6 months, except in high bleeding risk patients, for which 1–3 months DAPT may be preferred (Roffi et al., 2015; Ibanez et al., 2017). In ACS patients undergone coronary stenting, 12 months of DAPT (P2Y_12_ receptor antagonist between prasugrel or ticagrelor on top of aspirin lifelong) are recommended, whereas only 6 months of P2Y_12_ receptor antagonist treatment are warranted in addition to aspirin in high risk bleeding patients (Valgimigli et al., 2018).

Anticoagulant and antiplatelet combined therapy poses significant problems on the balance between efficacy and safety, which have been addressed in several studies (Oldgren et al., 2013). The “Anti-Xa therapy to lower cardiovascular events in addition to standard therapy in subjects with ACS-thrombolysis in myocardial infarction 51” (ATLAS-ACS 2 TIMI 51) trial has demonstrated 2.5 mg of rivaroxaban twice daily (low dose), on top of aspirin plus clopidogrel, to reduce the risk of death for cardiovascular causes, myocardial infarction, or stroke in non-AF patients with recent ACS, but to also increase the major bleeding and intracranial hemorrhage risks (Mega et al., 2012). Accordingly, a novel indication for triple antithrombotic therapy has been proposed, by the recent 2017 ESC guidelines for STEMI, in patients with ACS without AF or other specific indication for OAC, at low bleeding-risk (Ibanez et al., 2017). Regarding the AF setting, a cohort study on 82854 Danish AF patients with a mean age of 73.7 years has reported the risk of non-fatal and fatal bleeding in combination therapy (single or dual antiplatelet agents plus warfarin) to be at least threefold higher than monotherapy with warfarin (Hansen et al., 2010). Consistently, Hess et al. (2015) examined 4959 AF ≥65 years old patients undergoing PCI for MI: triple therapy produced higher rates of major bleeding than dual antithrombotic therapy, and no significative difference was measured in composite endpoint of MI, death, or stroke. Similar results have emerged from a recently published meta-analysis by Cavallari and Patti (2017).

## Guidelines and Consensus in Patients With AF Undergoing PCI

2016 ESC guidelines for AF recommend OAC monotherapy in AF patients with stable CAD and no PCI/ACS in the previous 12 months (Kirchhof et al., 2016). Regarding people requiring PCI, only little evidence exists, thus recommendations derive from a reviewed expert consensus (Lip et al., 2014) that proposes two different flowcharts: for elective PCI and for ACS; within them the physician has to assess the risk of cardiovascular event and stent thrombosis with respect to the risk of bleeding. Following these recommendations, triple therapy (OAC plus aspirin and clopidogrel) is provided for only 1 month except in patients undergoing PCI after ACS, for which it is extended to 6 months due to the high thrombotic risk. After this period and up to the 12th month since ACS or elective PCI, combination of single antiplatelet agent plus OAC is prompted, with the exception of the patients undergoing elective stenting at high bleeding risk, whose time of switch from double to single antiplatelet therapy is anticipated to 6 months. Subsequently, all patients have to continue single antithrombotic therapy with OAC lifelong (Kirchhof et al., 2016) in line with the AF ones, 2017 ESC guidelines for STEMI recommend the same therapeutic regimen (Ibanez et al., 2017). Differently, the 2016 Canadian Cardiovascular Society Guidelines for the management of AF propose no triple therapy for patients undergoing elective PCI, but combined therapy with OAC plus clopidogrel for 12 months, with a stratification of patients also based on age (above or below 65 years) (Macle et al., 2016). Finally, the Canadian Cardiovascular Society has recently published a Focused Update for the Use of Antiplatelet Therapy in which triple therapy with low dose OAC and DAPT (up to 6 months) is recommended for AF patients aged ≥65 years, undergoing PCI for ACS or high risk elective PCI (Mehta et al., 2018).

## New Evidences and Different Strategies: Woest, Pioneer AF-PCI and RE-DUAL PCI Trials

The latest reported evidence are partially based on the results of the “What is the Optimal antiplatElet and anticoagulant therapy in patients with OAC and coronary StenTing” (WOEST) trial, which has tested, in patients receiving oral anticoagulants and undergoing PCI, the efficacy and safety of clopidogrel alone (double therapy, 279 patients, mean age 70.3 years) compared with clopidogrel plus aspirin (triple therapy, 284 patients, mean age 69.5). The WOEST trial has shown double therapy to be safer than triple therapy and, surprisingly, even more effective (Dewilde et al., 2013). Regarding safety, which indeed constituted its primary outcome, and consistently with the previously mentioned Danish databases, the WOEST study confirms the benefit of double therapy on the incidence of total bleeding, compared with triple therapy. Conversely, due to the small simple size, which represents the main limitation of the study, the WOEST trial is not enough powerful to significantly detect difference in the secondary outcome of efficacy (reduction of the combined incidence of death, stroke, stent thrombosis, myocardial infarction and target vessel revascularization) (Rubboli and De Caterina, 2014). Anyhow, a meta-analysis by D’Ascenzo and collaborators has also highlighted no difference in subsequent cardiovascular events between therapy with clopidogrel plus OAC and triple antithrombotic therapy (D’Ascenzo et al., 2015). The major criticisms to the trial, in addition to the inadequate sample size, concern the exclusion criteria which do not allow the inclusion of patients at increased risk for stent thrombosis and the mean age of enrolled patients (about 70 years) that is lower than the real-world of unselected patients (about 73 years) (Rubboli and De Caterina, 2014).

In order to compare regimens of rivaroxaban with single or DAPT, the PIONEER AF-PCI trial has randomized 2124 NVAF patients undergone PCI with stenting into three groups in a 1:1:1 ratio. Group 1 (709 patients, mean age 70.4) received 15 mg rivaroxaban once daily plus P2Y_12_ receptor antagonist for 12 months; group 2 (709 patients, mean age 70.0) was treated with low dose rivaroxaban (2.5 mg twice daily) and DAPT (aspirin plus P2Y_12_ receptor antagonist) for 1, 6, or 12 months; standard therapy consisting of warfarin once daily plus DAPT (aspirin with P2Y_12_ receptor antagonist) for 1, 6, or 12 months was given to group 3 (706 patients, mean age 69.9). Regarding the primary safety endpoint, clinically significant bleeding rates were higher in the group receiving standard therapy than in the two rivaroxaban groups. No significant differences have been detected in incidence of cardiovascular death, stroke, MI, which constituted the secondary efficacy endpoint (Gibson et al., 2016). The same investigation group has afterwards published a *post hoc* analysis to report reduced risk of all-cause mortality or recurrent hospitalization for both rivaroxaban groups compared with standard therapy one (Gibson et al., 2017). It is important to note that, similar to the WOEST, the PIONEER trial was not powered for an efficacy outcome (Bhatt, 2016); furthermore, patients at very high stroke risk were excluded, as well as those with unknown cause anemia, creatinine clearance <30 mL/min, recent history of significant gastrointestinal bleeding (Alexopoulos et al., 2017). Although a fair percentage of ≥65 years aged patients in the trial, (more than 70% of entire population, with ≥75 years aged accounting to more than 33%) (Gibson et al., 2016), all the above mentioned exclusion criteria present in the trials are frequently found in the real world older patients. This point strongly limits a direct application of guidelines’ recommendations to this clinical setting and, in particular, the strict inclusion criteria of the PIONEER trial make its results not completely applicable to the geriatric population. A final perplexity derives from the primary composite endpoint of major/minor bleeding according to TIMI criteria, or bleeding requiring medical attention, that may have been inflated by the INR recommended range of 2–3, instead of 2–2.5 in the standard therapy group (Valgimigli et al., 2018).

**FIGURE 1:**
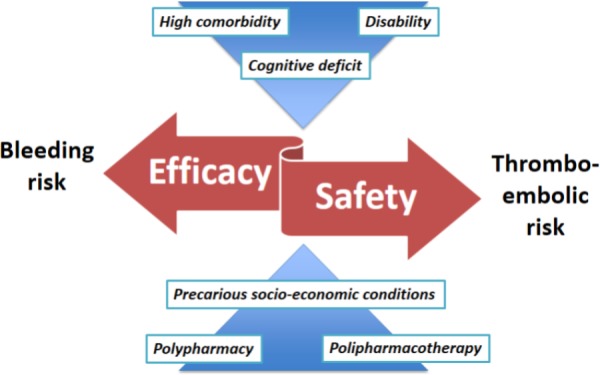
The impact of frailty on the critical balance between efficacy and safety in the management of antithrombotic therapy in the elderly.

However, despite the highlighted limits, PIONEER AF-PCI and WOEST have influenced, at least in part, the recommendation presented in the latest “2017 focused update on DAPT in CAD” from the ESC Task Force. Indeed, a relevant novelty has been introduced regarding patients undergoing PCI with indication for OAC: if bleeding overcomes ischemic risk, the possibility of not resorting to triple therapy is contemplated (Valgimigli et al., 2018). This latest indication is certainly of great interest for frail elderly patients; the proposed features of patient with an unfavorable profile for combination therapy with DAPT plus OAC seem to correspond to the characteristics of the frail elderly: advanced age, poor expected adherence, compromised mental status, end stage renal failure, history of major bleeding or hemorrhagic stroke, anemia (Cesari et al., 2014) (**Figure 1**). The latest mentioned ESC Task Force recommendations report a list of practical strategies to minimize bleeding complications in patients at high risk, including: periodically evaluate thrombotic and bleeding risks adopting validated risk scores, with particular attention to the modifiable factors; consider 2–2.5 INR target in case of warfarin therapy, which should be a second choice after NOACs agents; when a NOAC is prescribed, always use the lowest effective tested dose; prefer clopidogrel to prasugrel and ticagrelor as part of triple therapy (Valgimigli et al., 2018).

In the wake of the previously cited PIONEER AF-PCI, Cannon et al. (2017) have recently presented the results of the RE-DUAL PCI, a multicenter randomized trial whose target was to test the safety of dual therapy with dabigatran after PCI in AF patients, compared to triple therapy with warfarin. The authors have assigned 2725 patients in three 1:1:1 ratio randomized therapeutic regimes: dual therapy with 110 mg dabigatran twice daily plus P2Y_12_ receptor antagonist, dual therapy with 150 mg dabigatran twice daily and P2Y_12_ receptor antagonist, standard triple therapy with warfarin plus P2Y_12_ receptor antagonist and aspirin (aspirin was suspended after 1 month in patients implanted with bare-metal stent and after 3 months in patients implanted with drug-eluting stent). Due to recommendations of dabigatran label, elderly patients outside United States (≥80 years aged, except ≥70 years in Japan) had only been assigned to 110 mg dabigatran and triple therapy groups (Cannon et al., 2017). These dabigatran doses have previously been tested in the RE-LY trial, which has demonstrated them to provide stroke prevention in AF patients (Connolly et al., 2009). Regarding the primary safety outcome, major and clinically relevant bleeding according to the ISTH criteria, there is a substantial reduction of the incidence in both dual therapy groups compared to the triple therapy one. In particular, in the group treated with 110 mg dabigatran there was a reduction of bleeding of almost 50%, compared to the triple therapy, with statistical significance both in the non-inferiority and superiority tests. Similarly, the 150 mg dabigatran therapy has shown a reduction of 28% in bleedings compared to standard therapy, again with statistical significance both in non-inferiority and superiority tests; the results were consistent even considering the major bleeding or using TIMI bleeding criteria (Cannon et al., 2017). Concerning the secondary endpoint, both dabigatran dual-therapy regimes have shown non-inferiority to warfarin triple therapy in the composite efficacy endpoint (risk of death, thromboembolic events, unplanned revascularization) (Cannon et al., 2017; **Table 1**).

**Table 1:** Clinical trials on the antithrombotic management of patients with AF and CAD.

Clinical trial	Objective	Primary Endpoint	Results
*PIONEER AF-PCI* (Gibson et al., 2016) (2124 NVAF patients)	Compare the safety of 2 rivaroxaban treatment strategies^∗^ versus standard triple therapy through 12 months	TIMI clinically relevant bleeding (safety)	Rivaroxaban-based therapy was associated with a lower rate of clinically significant bleeding than standard triple therapy with VKA
*RE-DUAL PCI* (Cannon et al., 2017) (2725 NVAF patients)	Determine the safety of two dose dabigatran^†^ regimens versus standard triple therapy up to 30 months	ISTH clinically relevant bleeding (safety)	Dual therapy with both dabigatran doses was safer than standard triple therapy with VKA
*AUGUSTUS* (Bristol-Myers, 2018) (4600 NVAF patients)	Assess the non-inferiority safety of apixaban versus VKA (both combined with P2Y_12_ receptor antagonist) up to 6 months Demonstrate superiority safety of single antiplatelet therapy plus anticoagulant over triple therapy up to 6 months	ISTH clinically relevant bleeding (safety)	ONGOING (clinicaltrials.gov NCT02415400)
*ENTRUST AF-PCI* (Vranckx et al., 2017) (1500 NVAF patients)	Evaluate the safety of edoxaban plus P2Y_12_ receptor antagonist against warfarin and a P2Y_12_ receptor antagonist plus aspirin through 12 months	ISTH clinically relevant bleeding (safety)	ONGOING (clinicaltrials.gov NCT02866175)

^∗^*2.5 mg bis in die plus DAPT/15 mg rivaroxaban once daily;*^†^*110 mg twice daily/150 mg twice daily. ISTH, International Society of Thrombosis and Haemostasis; TIMI, Thrombolysis in Myocardial Infarction.*

With regard to age, elderly patients represented 16.8% of the entire population, whose mean age was 70.8 years, and the 110 mg dabigatran group (71.5 mean age) was composed of nearly 23% elderly. Interestingly, subgroup analysis revealed the advantage in the reduction of bleedings patients treated with 110 mg dabigatran to be substantially maintained in the elderly, whereas there was an increase in bleedings in the ≥80 years aged treated with the 150 mg dabigatran dose, compared to the triple therapy (Cannon et al., 2017). However, these results derived from a very limited number of patients, with only eight patients randomized to the 150 mg dabigatran group in the United States. Concerning thromboembolic events, there were no differences between the elderly and the young groups (Cannon et al., 2017).

Rubboli has conducted extensive analysis on the RE-DUAL PCI trial, bringing some interesting considerations: being aspirin interrupted in all cases at maximum 3 months since PCI, after that period the real comparison seems to turn into dabigatran 150 mg or 110 mg twice daily versus warfarin. With regard to the primary safety end-point, careful examination of the Kaplan–Meier curves reveals the two dabigatran curves in the 2-year chart to be precociously separated from the triple therapy one, particularly mostly during the first 3 months (Rubboli, 2017a). This remark may suggest the aspirin suspension to be the cause of the observed benefit. Furthermore, following the first 90 days, the gap between the 110 mg dabigatran and warfarin curves constantly increases, contrary almost the same difference for the whole period is maintained between the 150 mg dabigatran and the warfarin ones (Rubboli, 2017a). Otherwise, the observed safer effect on bleeding events of 110 mg dabigatran dose upon 150 mg one compared to warfarin is consistent with the results of the RE-LY trial (Connolly et al., 2009). Finally, it is surprising to note that the absolute incidence of stent thrombosis in the 110 mg dabigatran group is about twice the triple therapy one. This finding is difficult to interpret and cannot lead to any definitive conclusion due to the low number of observed events (15 vs. 8). It is possible to speculate that it may be due to the suboptimal efficacy of dual therapy, in particular in the case that the occurrence of stent thrombosis observed during the first 3 months. It would suggest triple therapy to be more effective in stent thrombosis prevention. Otherwise, considering the previously mentioned real comparison between dabigatran and warfarin after the first 3 months, if stent thrombosis events have been ascertained after the first 90 days, it may be hypothesized that the 110 mg twice-daily dabigatran dose is not adequate on this specific outcome but only the 150 mg dose. Indeed, the 150 mg twice daily dabigatran one has not shown difference in absolute incidence of stent thrombosis compared to warfarin triple therapy (Rubboli, 2017b).

## Ongoing Trials and Critical Points

Further studies are ongoing in AF patients undergoing PCI in order to compare standard triple therapy (DAPT plus warfarin) and dual therapy with NOAC plus P2Y_12_ receptor antagonist: AUGUSTUS trial (clinicaltrials.gov NCT02415400) is testing apixaban non-inferiority to warfarin on ISTH major or clinically relevant non-major bleeding in patients with concomitant P2Y_12_ receptor antagonist therapy. Moreover, this trial aims to test the superiority in safety of single antiplatelet therapy (P2Y_12_ receptor antagonist) plus anticoagulant (warfarin or apixaban) over anticoagulant plus DAPT (P2Y_12_ receptor antagonist plus aspirin) (Bristol-Myers, 2018), in order to better verify the effects related to cessation of aspirin, since its usefulness is uncertain and debated.

The same primary outcome will be tested in the ENTRUST AF-PCI trial (clinicaltrials.gov NCT02866175), which will randomize patients with AF undergoing PCI, to evaluate a treatment regimen based on the other direct factor Xa-inhibitor edoxaban once daily plus P2Y_12_ receptor antagonist (for 12 months) against warfarin and a P2Y_12_ receptor antagonist (for 12 months) plus aspirin (for 30 days to 12 months guided by clinical presentation, CHA_2_DS_2_-VASc and HASBLED scores) (Vranckx et al., 2017) (**Table 1**).

The two last mentioned trials will certainly contribute to better define timing and setting of combined antithrombotic therapies, as well as the choice of the safer and more effective OAC. Nevertheless, given the exclusion criteria, the geriatric population, especially the frail patients, are likely to result underrepresented, as systematically occurred in clinical trials (Cerreta et al., 2012; Bencivenga et al., 2017). AF and AMI are highly prevalent in >75 years aged population but the percentage of these patients included in ACS clinical trials is <7%, thus, only limited information exists on the benefit–risk balance (Andreotti et al., 2015). In this scenario, no surprise generates the lack of evidence-based recommendations for the frail population (Ridda et al., 2008).

Summarizing, according to the most relevant guidelines, personalization of antithrombotic therapies based on the balance between safety and efficacy, is nowadays allowed: AF patients undergoing PCI should be treated with triple therapy, for the shortest possible time, while dual therapy with single antiplatelet plus OAC may be preferred for patients at high bleeding risk. This latter option is especially important for older patients, which conversely generally more benefit of effective therapies than younger ones: if bleeding risk overcomes thrombotic one, the possibility of omitting ASA or P2Y_12_ inhibitors from the triple antithrombotic therapy since the time of elective coronary revascularization is now contemplated. In the next years, given the results of the ongoing trials, a possible therapeutic trend may be represented by association of single antiplatelet agent plus a NOAC at the lowest effective dose (lower than those approved for the treatment of AF) in the population at high bleeding risk, even for geriatric population.

In order to overcome the limits of current evidence and considering the unquestionable difficulty to include adequate sample size of elderly patients in antithrombotic therapy trials, it would be desirable to improve multicenter data registers, especially from centers specialized in the treatment of these subpopulations. In this way, data would originate from the “real world” of daily clinical practice, minimally restrictive regarding exclusion/inclusion criteria, with more flexible and patient-oriented treatment approaches. Further, mechanistic studies may be undertaken to help researchers test new approaches deriving from both clinical and laboratory characteristics. Given the increasing aging process of the overall population, the mentioned and other countermeasures are urgently needed.

## Conclusion

It is therefore clear how, in the last years, the scientific community has acknowledged the clinical relevance of the problematic “combined antithrombotic therapy” whose result was the publication of indications to support the work of the clinicians, no longer abandoned to their own experience. Although recent indisputable improvements in evidence and consensus, many points remain obscure or debated, and urgently require updates. Due to the inadequate power and the small number, to date the available clinical randomized trials do not always allow defining the optimal antithrombotic strategy for AF patients undergoing PCI, which necessarily needs to be still personalized.

As typical in clinical trials, the geriatric population, in particular the frail patient characterized by high comorbidity, disability, cognitive deficit, polypharmacy and precarious socio-economic conditions, is excluded from the large studies on which the guidelines are based. It would be desirable to improve multicenter register systems from centers specialized in the treatment of these subpopulations, and/or undertake mechanistic studies to help researchers test new approaches deriving from both clinical and laboratory data.

## Author Contributions

LB and GR conceived of the presented topic and performed the research of the most up-to-date evidence in the scientific literature. KK and GC verified the applied methods. NF encouraged LB to investigate the “elderly patient problem.” AC supervised the entire work. All authors discussed the key points and contributed to the final manuscript.

## Conflict of Interest Statement

The authors declare that the research was conducted in the absence of any commercial or financial relationships that could be construed as a potential conflict of interest.

## Abbreviations

ACS: acute coronary syndrome
AF: atrial fibrillation
CAD: coronary artery disease
DAPT: dual antiplatelet therapy
ESC: European Society of Cardiology
NOAC: new oral anticoagulation
NVAF: non-valvular atrial fibrillation
OAC: oral anticoagulation
PCI: percutaneous coronary intervention
VKA: vitamin K antagonist

## References

[B1] Alexopoulos D. Vlachakis P. Lekakis J. (2017). Triple antithrombotic therapy in atrial fibrillation patients undergoing PCI: a fading role. *Cardiovasc. Drugs Ther.* 31 319–324. 10.1007/s10557-017-6730-5 28643219

[B2] Andreotti F. Rocca B. Husted S. Ajjan R. A. ten Berg J. Cattaneo M. (2015). Antithrombotic therapy in the elderly: expert position paper of the European Society of Cardiology Working Group on Thrombosis. *Eur. Heart J.* 36:ehv304. 10.1093/eurheartj/ehv304 26163482

[B3] Bencivenga L. Grieco F. V. Femminella G. D. de Lucia C. Komici K. Rengo C. (2017). Management and treatment of cardiovascular diseases in the elderly. *Curr. Pharmacogenomics Person Med.* 15 1–9. 10.2174/1875692115666170508152820

[B4] Bhatt D. L. (2016). O PIONEERs! The beginning of the end of full-dose triple therapy with warfarin? *Circulation* 135 334–337. 10.1161/CIRCULATIONAHA.116.025923 27881554

[B5] Braun O. Bico Ö. Chaudhry U. Wagner H. Koul S. Tydén P. (2015). Concomitant use of warfarin and ticagrelor as an alternative to triple antithrombotic therapy after an acute coronary syndrome. *Thromb. Res.* 135 26–30. 10.1016/j.thromres.2014.10.016 25467434

[B6] Bristol-Myers S. (2018). *A Study of Apixaban in Patients With Atrial Fibrillation, Not Caused by a Heart Valve Problem, Who Are at Risk for Thrombosis (Blood Clots) Due to Having Had a Recent Coronary Event, Such as a Heart Attack or a Procedure to Open the Vessels of the Heart*. Available at: https://www.clinicaltrials.gov/ct2/show/NCT02415400

[B7] Cannon C. P. Bhatt D. L. Oldgren J. Lip G. Y. H. Ellis S. G. Kimura T. (2017). Dual antithrombotic therapy with dabigatran after PCI in atrial fibrillation. *N. Engl. J. Med.* 377:NEJMoa1708454. 10.1056/NEJMoa1708454 28844193

[B8] Cavallari I. Patti G. (2017). Meta-analysis comparing the safety and efficacy of dual versus triple antithrombotic therapy in patients with atrial fibrillation undergoing percutaneous coronary intervention. *Am. J. Cardiol.* 121 718–724. 10.1016/J.AMJCARD.2017.12.014 29373105

[B9] Cerreta F. Eichler H.-G. Rasi G. (2012). Drug policy for an aging population—The European medicines agency’s geriatric medicines strategy. *N. Engl. J. Med.* 367 1972–1974. 10.1056/NEJMp1209034 23171092

[B10] Cesari M. Gambassi G. van Kan G. A. Vellas B. (2014). The frailty phenotype and the frailty index: different instruments for different purposes. *Age Ageing* 43 10–12. 10.1093/ageing/aft160 24132852

[B11] Chugh S. S. Havmoeller R. Narayanan K. Singh D. Rienstra M. Benjamin E. J. (2014). Worldwide epidemiology of atrial fibrillation: a global burden of disease 2010 study. *Circulation* 129 837–847. 10.1161/CIRCULATIONAHA.113.005119 24345399 PMC4151302

[B12] Connolly S. J. Ezekowitz M. D. Yusuf S. Eikelboom J. Oldgren J. Parekh A. (2009). Dabigatran versus warfarin in patients with atrial fibrillation. *N. Engl. J. Med.* 361 1139–1151. 10.1056/NEJMoa0905561 19717844

[B13] D’Ascenzo F. Taha S. Moretti C. Omedè P. Grossomarra W. Persson J. (2015). Meta-analysis of randomized controlled trials and adjusted observational results of use of clopidogrel, aspirin, and oral anticoagulants in patients undergoing percutaneous coronary intervention. *Am. J. Cardiol.* 115 1185–1193. 10.1016/J.AMJCARD.2015.02.003 25799015

[B14] Dewilde W. J. Oirbans T. Verheugt F. W. Kelder J. C. De Smet B. J. Herrman J. P. (2013). Use of clopidogrel with or without aspirin in patients taking oral anticoagulant therapy and undergoing percutaneous coronary intervention: an open-label, randomised, controlled trial. *Lancet* 381 1107–1115. 10.1016/S0140-6736(12)62177-1 23415013

[B15] Gibson C. M. Mehran R. Bode C. Halperin J. Verheugt F. W. Wildgoose P. (2016). Prevention of bleeding in patients with atrial fibrillation undergoing PCI. *N. Engl. J. Med.* 375 2423–2434. 10.1056/NEJMoa1611594 27959713

[B16] Gibson C. M. Pinto D. S. Chi G. Arbetter D. Yee M. Mehran R. (2017). Recurrent hospitalization among patients with atrial fibrillation undergoing intracoronary stenting treated with 2 treatment strategies of rivaroxaban or a dose-adjusted oral vitamin K antagonist treatment strategy clinical perspective. *Circulation* 135 323–333. 10.1161/CIRCULATIONAHA.116.025783 27881555 PMC5266420

[B17] Giugliano R. P. Ruff C. T. Braunwald E. Murphy S. A. Wiviott S. D. Halperin J. L. (2013). Edoxaban versus warfarin in patients with atrial fibrillation. *N. Engl. J. Med.* 369 2093–2104. 10.1056/NEJMoa1310907 24251359

[B18] Granger C. B. Alexander J. H. McMurray J. J. V. Lopes R. D. Hylek E. M. Hanna M. (2011). Apixaban versus warfarin in patients with atrial fibrillation. *N. Engl. J. Med.* 365 981–992. 10.1056/NEJMoa1107039 21870978

[B19] Hansen M. L. Sørensen R. Clausen M. T. Fog-Petersen M. L. Raunsø J. Gadsbøll N. (2010). Risk of bleeding with single, dual, or triple therapy with warfarin, aspirin, and clopidogrel in patients with atrial fibrillation. *Arch. Intern. Med.* 170 1433–1441. 10.1001/archinternmed.2010.271 20837828

[B20] Hess C. N. Broderick S. Piccini J. P. Alexander K. P. Newby L. K. Shaw L. K. (2012). Antithrombotic therapy for atrial fibrillation and coronary artery disease in older patients. *Am. Heart J.* 164 607–615. 10.1016/j.ahj.2012.07.004 23067921 PMC3777661

[B21] Hess C. N. Peterson E. D. Peng S. A. de Lemos J. A. Fosbol E. L. Thomas L. (2015). Use and outcomes of triple therapy among older patients with acute myocardial infarction and atrial fibrillation. *J. Am. Coll. Cardiol.* 66 616–627. 10.1016/J.JACC.2015.05.062 26248987

[B22] Ibanez B. James S. Agewall S. Antunes M. J. Bucciarelli-Ducci C. Bueno H. (2017). ESC Guidelines for the management of acute myocardial infarction in patients presenting with ST-segment elevation. *Eur. Heart J.* 33 2569–2619. 10.1093/eurheartj/ehx393 22922416

[B23] Kirchhof P. Benussi S. Kotecha D. Ahlsson A. Atar D. Casadei B. (2016). ESC Guidelines for the management of atrial fibrillation developed in collaboration with EACTS. *Eur. Heart J.* 37 2893–2962. 10.1093/eurheartj/ehw210 27567408

[B24] Kirchner J. T. (1994). Nonsteroidal anti-inflammatory drug use in the elderly: issues of compliance and safety. *J. Am. Osteopath. Assoc.* 94 300–304. 8026998

[B25] Kralev S. Schneider K. Lang S. Süselbeck T. Borggrefe M. (2011). Incidence and severity of coronary artery disease in patients with atrial fibrillation undergoing first-time coronary angiography. *PLoS One* 6:e24964. 10.1371/journal.pone.0024964 21957469 PMC3177852

[B26] Lip G. Y. H. Windecker S. Huber K. Kirchhof P. Marin F. Ten Berg J. M. (2014). Management of antithrombotic therapy in atrial fibrillation patients presenting with acute coronary syndrome and/or undergoing percutaneous coronary or valve interventions: a joint consensus document of the European Society of Cardiology Working Group on. *Eur. Heart J.* 35 3155–3179. 10.1093/eurheartj/ehu298 25154388

[B27] Macle L. Cairns J. Leblanc K. Tsang T. Skanes A. Cox J. L. (2016). Focused update of the Canadian cardiovascular society guidelines for the management of atrial fibrillation. *Can. J. Cardiol.* 32 1170–1185. 10.1016/j.cjca.2016.07.591 27609430

[B28] Mant J. Hobbs F. R. Fletcher K. Roalfe A. Fitzmaurice D. Lip G. Y. (2007). Warfarin versus aspirin for stroke prevention in an elderly community population with atrial fibrillation (the Birmingham Atrial Fibrillation Treatment of the Aged Study, BAFTA): a randomised controlled trial. *Lancet* 370 493–503. 10.1016/S0140-6736(07)61233-1 17693178

[B29] Mega J. L. Braunwald E. Wiviott S. D. Bassand J.-P. Bhatt D. L. Bode C. (2012). Rivaroxaban in patients with a recent acute coronary syndrome. *N. Engl. J. Med.* 366 9–19. 10.1056/NEJMoa1112277 22077192

[B30] Mehta S. R. Bainey K. R. Cantor W. J. Lordkipanidzé M. Marquis-Gravel G. Robinson S. D. (2018). Canadian cardiovascular society/Canadian association of interventional cardiology focused update of the guidelines for the use of antiplatelet therapy. *Can. J. Cardiol.* 34 214–233. 10.1016/j.cjca.2017.12.012 29475527

[B31] Oldgren J. Wallentin L. Alexander J. H. James S. Jonelid B. Steg G. (2013). New oral anticoagulants in addition to single or dual antiplatelet therapy after an acute coronary syndrome: a systematic review and meta-analysis. *Eur. Heart J.* 34 1670–1680. 10.1093/eurheartj/eht049 23470494 PMC3675388

[B32] Patel M. R. Mahaffey K. W. Garg J. Pan G. Singer D. E. Hacke W. (2011). Rivaroxaban versus warfarin in nonvalvular atrial fibrillation. *N. Engl. J. Med.* 365 883–891. 10.1056/NEJMoa1009638 21830957

[B33] Ridda I. Lindley R. MacIntyre R. C. (2008). The challenges of clinical trials in the exclusion zone: the case of the frail elderly. *Australas. J. Ageing* 27 61–66. 10.1111/j.1741-6612.2008.00288.x 18713194

[B34] Roffi M. Patrono C. Collet J.-P. Mueller C. Valgimigli M. Andreotti F. (2015). ESC Guidelines for the management of acute coronary syndromes in patients presenting without persistent ST-segment elevation. *Eur. Heart J.* 37 267–315. 10.1093/eurheartj/ehv320 26320111

[B35] Rubboli A. (2017a). Superior safety of dual therapy with dabigatran and clopidogrel vs. triple therapy with warfarin, aspirin and clopidogrel in the RE-DUAL PCI trial: What is key, the strategy or the drug? *Eur. J. Intern. Med.* 46 e40–e41. 10.1016/J.EJIM.2017.10.001 28986160

[B36] Rubboli A. (2017b). What is the significance, if any, of the increased incidence of stent thrombosis with dual therapy of dabigatran 110 mg twice daily and clopidogrel in the RE-DUAL PCI trial? *Eur. J. Intern. Med.* 50 e27–e28. 10.1016/j.ejim.2017.12.009 29277476

[B37] Rubboli A. De Caterina R. (2014). The WOEST study: critical considerations and applicability. *Cor Vasa* 56 e254–e258. 10.1016/J.CRVASA.2014.03.001

[B38] Sarafoff N. Martischnig A. Wealer J. Mayer K. Mehilli J. Sibbing D. (2013). Triple therapy with aspirin, prasugrel, and vitamin K antagonists in patients with drug-eluting stent implantation and an indication for oral anticoagulation. *J. Am. Coll. Cardiol.* 61 2060–2066. 10.1016/j.jacc.2013.02.036 23524219

[B39] Schmitt J. Duray G. Gersh B. J. Hohnloser S. H. (2009). Atrial fibrillation in acute myocardial infarction: a systematic review of the incidence, clinical features and prognostic implications. *Eur. Heart J.* 30 1038–1045. 10.1093/eurheartj/ehn579 19109347

[B40] Sharma M. Cornelius V. R. Patel J. P. Davies J. G. Molokhia M. (2015). Efficacy and harms of direct oral anticoagulants in the elderly for stroke prevention in atrial fibrillation and secondary prevention of venous thromboembolism: systematic review and meta-analysis. *Circulation* 132 194–204. 10.1161/CIRCULATIONAHA.114.013267 25995317 PMC4765082

[B41] Valgimigli M. Bueno H. Byrne R. A. Collet J.-P. Costa F. Jeppsson A. (2018). 2017 ESC focused update on dual antiplatelet therapy in coronary artery disease developed in collaboration with EACTS: the task force for dual antiplatelet therapy in coronary artery disease of the European Society of Cardiology (ESC) and of the European Association for Cardio-Thoracic Surgery (EACTS). *Eur. Heart J.* 39 213–260. 10.1093/eurheartj/ehx419 28886622

[B42] Vranckx P. Lewalter T. Valgimigli M. Tijssen J. G. Reimitz P.-E. Eckardt L. (2017). Evaluation of the safety and efficacy of an edoxaban-based antithrombotic regimen in patients with atrial fibrillation following successful percutaneous coronary intervention (PCI) with stent placement: rationale and design of the ENTRUST-AF PCI trial. *Am. Hear J.* 196 105–112. 10.1016/j.ahj.2017.10.009 29421002

[B43] Wilke T. Groth A. Mueller S. Pfannkuche M. Verheyen F. Linder R. (2013). Incidence and prevalence of atrial fibrillation: an analysis based on 8.3 million patients. *Europace* 15 486–493. 10.1093/europace/eus333 23220354

